# Early percutaneous coronary intervention and in-hospital mortality in non st elevation coronary syndromes

**DOI:** 10.1186/2197-425X-3-S1-A750

**Published:** 2015-10-01

**Authors:** J Cebrián Domenech, MT Gisbert Garcia, K Vacacela Cordova, L De Hevia Benlliure, I Madrid Lopez, C Calabuig Guillem, M Piñol Ribas, R Gimeno Costa, J Bonastre Mora, A Castellanos Ortega

**Affiliations:** Hospital Universitario y Politécnico La Fe, ICU, Valencia, Spain

## Introduction

PCI timing remains controversial in patients with non ST elevation coronary syndromes.

## Objectives

To analyze the influence of early PCI (ePCI) on in-hospital mortality in this population.

## Methods

We analyzed 449 patients with diagnosis of non ST elevation coronary syndrome consecutively admitted in our teaching referrall ICU during the period 01/01/2012 - 20/03/2015. When PCI was performed during ICU it was considered ePCI. Otherwise (patients without PCI or with delayed PCI after ICU stay) were considered non ePCI. We analyzed the influence of ePCI in hospital mortality. An univariate analysis with in-hospital mortality as the main outcome and NYHA, Saps 3, age and sex as the main covariates was performed. A binary logistic regression was also performed to adjust for the main confounders. Chi square, T-test and binary logistic regression were used.

## Results

A total of 296 patients were treated with ePCI. Those with ePCI had a lower mortality than those without ePCI (4.07% vs 9.15%; p 0.029). The multivariate analysis showed that the independent predictors were both Saps 3 and NYHA score. Results of both univariate and multivariate analysis are shown in Tables 1 and 2.

**Table 1:** Univariate Analysis. ePCI patiens were younger and with lower severity scores than their counterparts without ePCI.

Variable	With ePCI	Without ePCI	p
N	229	220	
**Age (years)**	65.7 (SD 12.7)	70.4 (SD 12.0)	0.001
**Female Sex (%)**	21.96	32.68	0.014
**Saps 3 score**	44.78 (SD 7.80)	47.81 (SD 8.92)	0.001
**NYHA (III) (%)**	6.42	13.73	0.001
**In-Hospital Mortality (%)**	4.07	9.15	0.029

**Table 2:** Multivariate Analysis.

Variable	Odss Ratio	95% CI	p
**Saps 3 score**	1.13	1.08-1.18	0.001
**NYHA II**	2.94	0.91-9.45	0.071
**NYHA III**	5.88	1.45-23.84	0.013
**ePCI**	0.59	0.26-1.36	0.218

After multivariate analysis ePCI lost his protective effect and mortality seems to be mainly related to patient´s severity (saps 3 or NYHA score)

## Conclusions

In our population ePCI do not determine in-hospital mortality. Severity measured either by Saps 3 or by NYHA scores, and not ePCI, seems to be the major determinant of mortality.

